# *Pediococcus acidilactici* reduces tau pathology and ameliorates behavioral deficits in models of neurodegenerative disorders

**DOI:** 10.1186/s12964-023-01419-3

**Published:** 2024-01-30

**Authors:** Yong Zhang, Weiyi Qian, Yitong Zhang, Yan Ma, Jiamin Qian, Jinping Li, Xun Wei, Yan Long, Xiangyuan Wan

**Affiliations:** 1 Shunde Innovation School, Research Institute of Biology and Agriculture, University of Science and Technology Beijing, Beijing, 100083 China; 2 Zhongzhi International Institute of Agricultural Biosciences, Beijing, 100083 China; 3 Beijing Engineering Laboratory of Main Crop Bio-Tech Breeding, Beijing International Science and Technology Cooperation Base of Bio-Tech Breeding, Beijing Solidwill Sci-Tech Co. Ltd, Beijing, 100192 China

**Keywords:** Brain iron accumulation, Gut-brain axis, *Pediococcus acidilactici*, Tau reducing, AP activity

## Abstract

**Background:**

Alzheimer’s disease (AD), affecting many elders worldwide, is characterized by A-beta and tau-related cognitive decline. Accumulating evidence suggests that brain iron accumulation is an important characteristic of AD. However, the function and mechanism of the iron-mediated gut-brain axis on AD is still unclear.

**Methods:**

A *Caenorhabditis elegans* model with tau-overexpression and a high-Fe diet mouse model of cognitive impairment was used for probiotic function evaluation. With the use of qPCR, and immunoblotting, the probiotic regulated differential expression of AD markers and iron related transporting genes was determined. Colorimetric kits, IHC staining, and immunofluorescence have been performed to explore the probiotic mechanism on the development of gut-brain links and brain iron accumulation.

**Results:**

In the present study, a high-Fe diet mouse model was used for evaluation in which cognitive impairment, higher A-beta, tau and phosphorylated (p)-tau expression, and dysfunctional phosphate distribution were observed. Considering the close crosstalk between intestine and brain, probiotics were then employed to delay the process of cognitive impairment in the HFe mouse model. *Pediococcus acidilactici* (PA), but not *Bacillus subtilis* (BN) administration in HFe-fed mice reduced brain iron accumulation, enhanced global alkaline phosphatase (AP) activity, accelerated dephosphorylation, lowered phosphate levels and increased brain urate production. In addition, because PA regulated cognitive behavior in HFe fed mice, we used the transgenic *Caenorhabditis elegans* with over-expressed human p-tau for model, and then PA fed worms became more active and longer lived than *E.coli* fed worms, as well as p-tau was down-regulated. These results suggest that brain iron accumulation influences AD risk proteins and various metabolites. Furthermore, PA was shown to reverse tau-induced pathogenesis via iron transporters and AP-urate interaction.

**Conclusions:**

PA administration studies demonstrate that PA is an important mediator of tau protein reduction, p-tau expression and neurodegenerative behavior both in *Caenorhabditis elegans* and iron-overload mice. Finally, our results provide candidates for AP modulation strategies as preventive tools for promoting brain health.

Video Abstract

**Supplementary Information:**

The online version contains supplementary material available at 10.1186/s12964-023-01419-3.

## Background

Alzheimer’s disease (AD) is the most common type of neurodegenerative dementia, affecting more than 50 million people worldwide. It robs people of their physical independence, and has become the fifth leading cause of death resulting from global population aging [1]. Its pathogenic mechanisms have been described by different hypotheses. Over the past decade, several different drugs have been developed for AD; however, almost all compounds have failed in clinical studies and only a few have attenuated the progression of the disease [2]. Accumulating evidence indicates that brain iron accumulation in AD is positively associated with cognitive deterioration via oxidative stress, ferroptotic cell death and related inflammation [3]. Although such clinical phenomena reflect the role that iron plays in the nervous system triggering memory damage, further mechanisms and treatments remain unknown.

Iron homeostasis is precisely manipulated from gut to brain and involves a vast network of metabolic regulation. Iron (Fe^2+^) is mainly transported by importing divalent metal transporter 1 (DMT1) and exporting transporter ferroportin (Fpn) on the intestinal epithelium, while circulating iron is transformed into Fe^3+^ bound to transferrin (TF). Cytochrome B reductase 1 (CYBRD1) has recently been discovered to be solely responsible for duodenal iron homeostasis where iron is reduced to Fe^2+^ across the cell membrane [4]. In particular, iron accumulation in the brain is accomplished through a constant exchange among the transferrin system on the blood-brain barrier (BBB), neurons, microglia and astrocytes [5]. Excess iron can facilitate the progression of oxidative stress, trigger aging and neurodegeneration. Furthermore, brain iron overload leads to the accumulation of neurodegenerative microtubule-associated protein tau [6], β-amyloid precursor protein (APP) [7], TAR DNA binding protein 43 (TDP-43) [8] and osteopontin (OPN) [9]. However, the mechanism underlying iron pathobiology in neurodegenerative diseases is not fully understood.

Converging evidence suggests that iron homeostasis and related metabolic effects in the intestine are regulated by gut microbiota [10]. Iron overload in the gut and duodenum lead to selection on specific bacterial groups (such as *Lactobacillus reuteri*) that produce metabolites which affect the expression of hypoxia inducible factor 2 subunit alpha (HIF-2α) and iron transporters [11]. More importantly, A-beta peptide-mediated neuronal apoptosis can be regulated by iron-binding protein lipocalin-2 (LCN2) and its receptor SLC22A17 [12], which participate in regulating inflammatory cytokines and iron accumulation in the brain [13]. Nonetheless, how iron overload and altered microbiota influence the brain is largely unknown. Recently, the idea of microbiome-based therapeutics and exploration of the probiotic-regulated gut-brain axis have received much attention [14]. Some probiotics prevent iron damage in vivo and even improve neurodegeneration in mice, although there is little evidence regarding their role in human dementia [15–17]. Thus, our hypothesis is that microbial intervention can alleviate cognitive impairment caused by brain iron accumulation in an animal model via crosstalk between the intestine and brain.

In this study, we evaluated whether our screened probiotic strains alleviate brain iron accumulation in an HFe-fed mouse model and explored the mechanism of their anti-neurodegenerative effect. Previous probiotic studies have reported the lifespan-extending effect of *Pediococcus acidilactici* (PA) from pickles and the fat pad-reducing ability of strain *Bacillus subtilis* noon (BN) from fermented noon bean product [18, 19]. Here, we investigated whether administration of these two probiotics could prevent AD-like memory loss associated with brain iron accumulation in mice. In addition, we used the nematode *Caenorhabditis elegans* (*C.elegans*) because of its short life span, transparent body, simple symbiotic system, easily recognizable behavior and presence of most iron transporter genes to provide further mechanical validation of the role of p-tau hyperphosphorylation in AD-like impairment and to test the ability of probiotics to lower p-tau in the rodent model. In addition, related gene expression and metabolites altered by PA intake were identified in mice and validated in nematodes. The results from our in vivo studies provide important insights into the pathobiology of iron’s role in AD and its associated metabolic effects.

## Results

### PA improves cognitive performance in HFe-fed mice

**Fig. 1:**
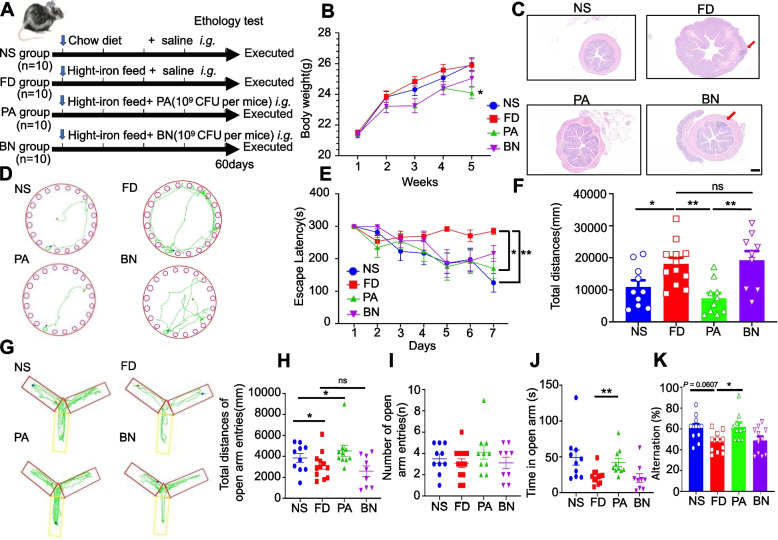
Improvement by PA intervention of spatial learning and memory and intestinal histology after iron overload. **A** Study design. The animals were randomly separated into four groups (*n* = 10 per group) 8 weeks after birth. The FD, PA, BN groups received the HFe diet for 60 days, whereas the NS group received a normal diet. **B** Body weights of NS, FD and probiotic-treated mice during the experimental protocol (10 mice per group). One-way ANOVA with post-hoc LSD test was used for significance. **C** Representative histological images of colon tissue from the NS control group and FD, PA and BN-treated mice by H&E staining of colon samples. The red arrows represent the muscular layer with inflammatory lymphatic infiltration. **D** Trajectory map of mice entering the Barnes mazes during the positioning navigation test. The green circle represents the final escape box, and the green line represents the entire escape path of the mouse. **E** Evasion latency of mice in positioning navigation experiments over 7 consecutive days of experimentation. Each point represents the mean escape time for each group. One-way ANOVA with post-hoc LSD test was used for significance. **F** Distance travelled by the mice (10 mice per group) on the last day of the experiment (day 7) to locate the escape box. Each point represents the total distance the mouse navigated. One-way ANOVA with post-hoc LSD test was used for significance. **G** Trajectory map of mice entering the Y-maze after novel arm opening. The red rectangle represents the start arm area, the yellow represents the novel arm area, and the green line represents the mouse exploration path. **H** Total distances that the mice (10 mice per group) explored after novel arm entry. Each point represents the total distance each mouse navigated. One-way ANOVA with post-hoc LSD test was used for significance. **I** Total number of times that mice (10 mice per group) entered the novel arm during the exploration period. One-way ANOVA with post-hoc LSD test was used for significance. **J** Total time spent by the mice (10 mice per group) in the novel arm during Y-maze exploration. One-way ANOVA with post-hoc LSD test was used for significance. **K** The percent of right choices in mice(10 mice per group) during Y-maze exploration. One-way ANOVA with post-hoc LSD test was used for significance. All data are means ± SEM. Statistical significance vs. the vehicle-only treatment group. *p < 0.05, **p < 0.01, ***p < 0.001

To explore the role of probiotics in cognitive impairment, we established a high-Fe diet (HFe) mouse model. Considering that the gut microbiota can be regulated by probiotics, we fed mice an HFe diet for 60 days and used BN or PA in the probiotic intervention groups beginning in the first week (Fig. 1A). Behavioral experiments were performed 1 week prior to euthanatization. Body weights of the mice showed that the groups started with an even weight distribution, but after 6 weeks of the diet the weights of the probiotic groups were significantly lower compared to control animals (Fig. 1B). Because the iron diet may also induce colonic inflammation [20], we also prepared H&E sections of the colon. The colonic muscle layer of FD mice was thickened, with severe inflammatory lymphoid infiltration and destruction of intestinal epithelial structure, while the muscle layer of mice in the NS group was thinner and structurally intact (Fig. 1C). The PA group exhibited structural integrity with a thickened muscle layer and minor lymphatic infiltration, while the BN group had a thickened muscular layer and showed less inflamed lymphatic infiltration than the FD group (Fig. 1C). The histological scores of colitis were significantly higher in the FD group than in the NS and PA group, but were not higher in the BN group (Supplemental Fig. 1A).

Next, we tested the learning ability of the mice using the Barnes maze task and Y-maze test. The Barnes maze test showed no difference between the BN and PA treatment groups in the beginning (1 day), but was different at the end of the training period (7 days; Fig. 1D). The PA group showed significant learning and escape ability, as revealed by decreases in the primary escape latency. However, there was also an initial display of spatial learning in the BN group which was different from that of the FD mice; however, this effect was diminished at a later stage in the BN group (7 days; Fig. 1E). In addition, the escape distance required by the mice was significantly reduced in the PA group (Fig. 1F).

To determine whether PA and BN could improve short-term memory in the HFe diet model, Y-maze learning was also evaluated in this experiment. Positive data was obtained from the Y-maze spontaneous alternation task test (Fig. 1G-J). This further substantiated the significant improvement in the PA group for spatial learning and working memory compared to the FD group. PA intake significantly increased the distance and residence time of the mice when exploring in the novel arm but had no effect on the number of times they entered the novel arm (Fig. 1H-J). Besides, PA intake significantly increased the percent of right choices in mice compared to the FD group (Fig. 1K). However, the BN group did not show any significant difference in these tests; hence, we did not explore the underlying mechanism of BN in the following experiments.

### PA decreased AD-associated tau and TDP43 protein content in mice and *C.elegans*

**Fig. 2:**
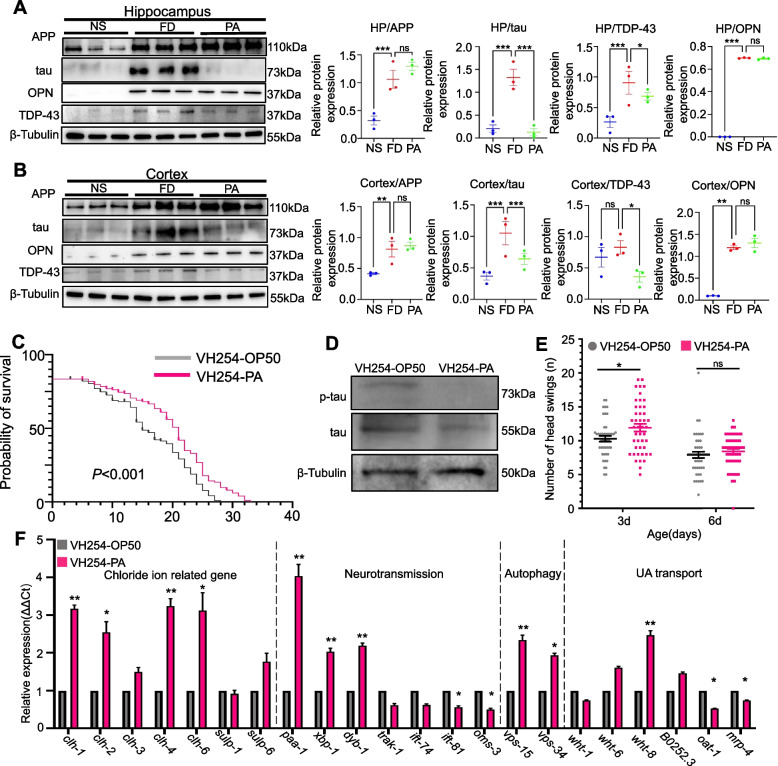
Markers modified by PA intervention and the tau-reducing effect in mice and * C. elegans.* **A** APP, tau, OPN and TDP43 proteins in hippocampal tissue. Western blotting analysis of NS, FD and PA-treated groups (3 mice per group). Quantification of western blotting grayscale values for APP, tau, OPN and TDP43 relative to β-tubulin in the hippocampus analyzed by Image J.One-way ANOVA with post-hoc LSD test was used for significance. **B** APP, tau, OPN and TDP43 proteins in cortical tissue using western blotting analysis of the NS-, FD- and PA-treated groups (3 mice each group). Quantification of western blotting grayscale values for APP, tau, OPN and TDP43 relative to β-tubulin in the prefrontal cortex analyzed by Image J. One-way ANOVA with post-hoc LSD test was used for significance. **C** Comparison of PA and *E. coli* OP50 on the lifespans of *C.elegans* VH254 (120 worms each group). Paired t-tests were used for significance. **D** Western blotting of tau and p-tau protein in PA- or *E. coli* OP50-treated *C.elegans* VH254 (700 worms each group). Paired t-tests were used. **E** Number of head swings in *C. elegans* VH254 after PA or in OP50 controls (above 40 worms each group). Paired t-tests were used for significance. **F** Expression of *clh1*, *clh2*, *clh3*, *clh4*, *clh6*, *sulp1*, *sulp6*, *paa-1*, *xbp-1*, *dyb-1*, *rak-1*,*ift74*, *ift81*, *osm-3*, *vps15*, *vps34*, *wht1*, *wht6*, *wht8*, *B0252.3*, *oat1* and *mrp4* relative to TBA-1 between PA and OP50 groups (600 worms each group). Paired t-tests were used for significance. All data are means ± SEM. Statistical significance vs. the vehicle-only treatment group. *p < 0.05, **p < 0.01, ***p < 0.001, ns: no significant difference

We next investigated the relationship between behavioral alterations and probiotic-regulated neuronal activation in different brain regions. We focused on some major neurodegenerative protein markers including APP, tau, OPN and TDP43. Firstly, APP aggregation occurred in brain tissues under the influence of the HFe diet. Interestingly, PA treatment promoted tau protein degradation and attenuated TDP43 protein content in the hippocampus and prefrontal cortex but had no effect on APP and OPN protein expression (Fig. 2A, B; Supplemental Fig. 1B, 5 mice per group). Moreover, we found that tau protein in the hippocampus was slightly enhanced compared to the prefrontal cortex by PA administration. Furthermore, OPN was upregulated in the hippocampus and prefrontal cortex with HFe diet intake (Fig. 2A, B).

To further confirm the role of PA in tau protein’s function, we analyzed longevity, p-tau expression and the behavior of p-tau-transgenic *C. elegans* after PA or OP50 intake. Compared to OP50 control, there was a significantly longer lifespan of worms after PA administration (Fig. 2C, *p* < 0.01, 120 worms per group). To determine whether probiotic-mediated tau protein toxicity was involved, a western-blotting experiment was used. The results showed that tau and p-tau protein were down-regulated after PA intervention (Fig. 2D, 700 worms per group). PA also notably enhanced the head swing behavior compared to OP50 controls but had no influence on pumping rates or body bends, suggesting an improvement in motor actions of head by PA (Fig. 2E, *p* < 0.01, 45 worms per group; Supplemental Fig. 1B).

Moreover, we analyzed mRNA expression to investigate the systemic response to PA in p-tau-transgenic nematodes (Fig. 2F, *p* < 0.01, 600 worms per group). PA promoted the expression of the chloride ion-related genes, *clh1* (3-fold), *clh2* (2.5-fold), *clh4* (3-fold), *clh6* (3-fold) and *sulp6* (2-fold). Importantly, PA induced a 4-fold increase in *paa-1* mRNA levels, which is responsible for tau dephosphorylation. Moreover, the transcriptional level of *xbp-1*, which can degrade mis-folded tau accumulation in the endoplasmic reticulum of *C.elegans* [18], was significantly increased by PA intervention. In addition, PA intake resulted in an elevated mRNA level of *dyb-1*, an enhancer of the tau-induced Unc phenotype, which contributes to neurotransmission [21]. Considering the axonal transport of tau, molecular motor transportation contributed to the mislocalization and accumulation of tau [22]. Interestingly, molecular motor-transporting *osm3* and *ift-81* mRNA levels were significantly decreased by PA. Another important effect of PA was to upregulate the transcripts *vps15* and *vps34*, encoding proteins involved in the formation of the autophagosome membrane [23], which is consistent with bec-1-mediated autophagy enhanced by PA in the hippocampus of mice. In addition, PA significantly upregulated UA transporter *wht8* (homolog to ABCG2) and decreased the UA excretion gene *oat-1* (homolog to OAT1) in p-tau-transgenic nematodes [24].

### PA plays different roles in duodenum and colon tissue under the HFe diet

**Fig. 3:**
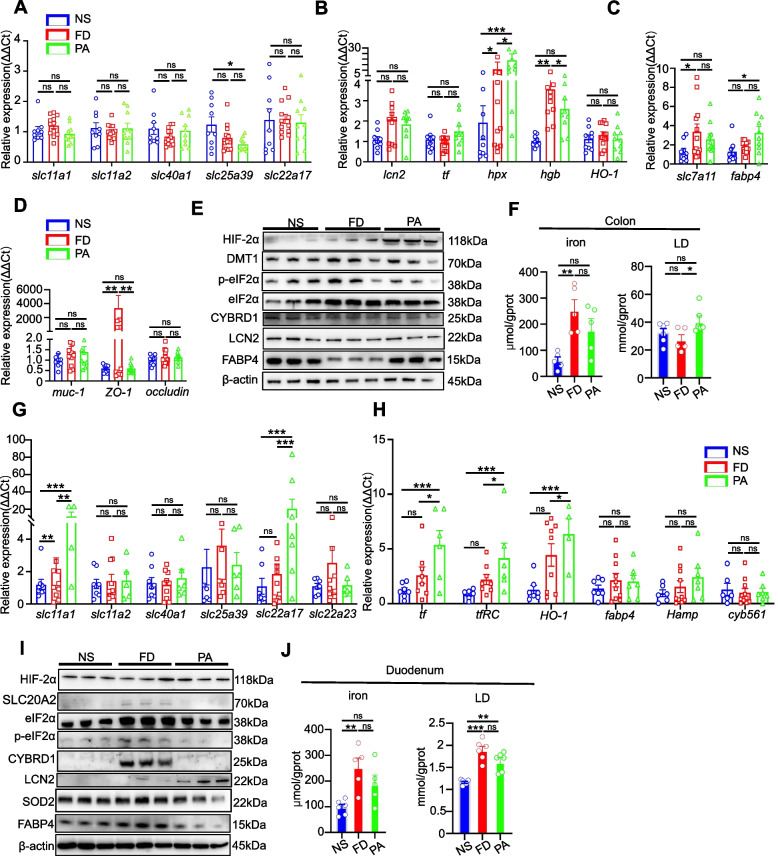
Effects of PA on colon and duodenum in HFe diet mice. **A** mRNA expression of iron transporter genes in the colon in NS-, FD-, and PA-treated groups (above 8 mice each group).One-way ANOVA with post-hoc LSD test was used for significance. **B** mRNA expression of iron-binding protein genes or metabolic genes in the colon of NS-, FD- and PA-treated groups (above 8 mice each group).One-way ANOVA with post-hoc LSD test was used for significance. **C **mRNA expression of *slc7a11* and *fabp4* genes in the colon of NS-, FD- and PA-treated groups (above 8 mice each group).One-way ANOVA with post-hoc LSD test was used for significance. **D** mRNA expression of gut tight junction function-related genes in NS-, FD- and PA-treated groups (above 8 mice each group).One-way ANOVA with post-hoc LSD test was used for significance. **E** Western blotting showing the effects of PA on the expression levels of proteins involved in iron transport, cell stress and metabolic regulation in mouse colonic tissue (3 mice each group). **F** Comparison of iron and LD levels in colonic tissue (above 5 mice each group). One-way ANOVA with post-hoc LSD test was used for significance. **G** mRNA expression of iron transporter genes of the duodenum in NS-, FD- and PA-treated groups (above 8 mice each group). One-way ANOVA with post-hoc LSD test was used for significance. **H** Expression of iron-binding protein genes or related genes in the duodenum of NS-, FD- and PA-treated groups (above 8 mice each group). Paired t-tests were used for significance. **I** Western blotting showing the effects of PA on levels of proteins involved in iron transport, cell stress and metabolic regulation in the duodenal tissue from the mice (3 mice each group). **J** Quantification of iron and LD levels in duodenal segments (above 5 mice each group). Paired t-tests were used for significance. All data are means ± SEM. Statistical significance vs. the vehicle-only treatment group. *p < 0.05, **p < 0.01, *** < 0.001, ns: no significant difference

It is well-established that probiotics have a strong effect on gut epithelium and microbiota, which affects diverse biological functions. For iron related proteins, *tf* (transferrin) is the major iron combined form in body, and iron transporters (*lcn2*, *slc11a1* and *Dmt1)* play different roles [25]. Another form of iron is heme, and transporters slc25a39, *hgb, hgx* and *ho-1* play different roles [25]. We firstly quantified expression of the main iron transporters (*lcn2*, *slc11a1* and *Dmt1*) in the colon by q-PCR analysis. We confirmed that iron mainly bound to *tf* (transferin) and then was intracellularly transported via *slc11a1* and *Dmt1* [26, 27]. However, there was no significant difference between the relative mRNA levels of iron transporters in colons of the NS, FD and PA groups, with the exception of *slc25a39* (NS vs PA, *p* < 0.05) (Fig. 3A), indicating that iron overload transport did not occur in the colon. slc25a39 is responsible for mitochondrial glutathione uptake in heme biosynthesis [28]. Furthermore, *lcn2* and *tf*, which have been characterized as iron binding protein and iron carriers in systemic circulation, did not show a significant difference (*p* > 0.05, Fig. 3B). In addition, *hgb* was upregulated by the HFe diet (NS vs FD, *p* < 0.05) and PA reduced *hgb* expression significantly (FD vs PA, *p* < 0.05), but *ho-1* was not modified (Fig. 3B). This suggests that iron overload selectively favors the iron-containing heme in the colon since *hpx* contributes to transporting heme and hgb produces heme [29]. Interestingly, expression of the fatty acid binding protein gene *fabp4* was increased by the HFe diet (NS vs FD, *p* < 0.05), but was not increased significantly in the PA group (FD vs PA, *p* > 0.05), whereas ferroptosis-related amino acid transporter *slc7a11* in the PA group showed a tendency to be reduced compared to the significant rise in expression in the FD group (NS vs FD, *p* < 0.05; Fig. 3C). In addition, to explore the effects of iron overload and PA intake on the intestinal barrier function, expression of genes *zo-1*, *muc-1* and *occludin* were examined. The results showed that PA reduced the expression of *zo-1* but had no effect on the adherent junction protein *muc-1* or the tight junction protein occludin (Fig. 3D).

It is widely known that gut bacteria influence host iron absorption by affecting HIF-2α [10]. In the present study, PA consumption tended to decrease colonic iron concentration while elevating HIF-2α protein amount in iron-overloaded mice (Fig. 3E). Other iron modulating proteins, such as DMT1, LCN2 and CYBRD1, were not differentially expressed at the protein level in the colon (Fig. 3E). The expression content of FABP4 was restored to normal levels in the PA group (Fig. 3F). Previous research has revealed that the interaction of HIF-2α with bacteria results in lactate production (LD) [30], a phenomenon we also observed in this study (Fig. 3F). However, the expression of the mitochondrial stress molecule eukaryotic translation Initiation factor 2A (eIF2α) and phosphorylation of eIF2α (p-eIF2α) was not influenced in the PA group (Fig. 3E).

Particularly, the duodenum, as a key site for absorption and storage of various metal ions, is also crucial in iron homeostasis [10]. Interestingly, the expression of iron transporters *slc11a2* and *slc40a1* was not significantly different between the NS, FD and PA groups, whereas *slc11a1* and *slc22a17* showed striking upregulation in the PA groups (Fig. 3G). Furthermore, other iron binding protein such as *tf*, *trRC* and *HO-1* were more highly expressed in the PA group (Fig. 3H). In contrast, other iron-related transcripts in the duodenum, such as *slc25a39*, *slc22a23*, *Hamp* and *cyb561* did not show any difference (Fig. 3G-H). Because of the high expression of receptor SLC22A17, we observed high LCN2 expression in the PA group (Fig. 3I). PA also showed a trend of reducing iron concentration and LD level in the duodenum (Fig. 3J). The above results showed that PA may have different effects on different sites to maintain iron homeostasis.

### PA protects mouse brain under a HFe diet

**Fig. 4:**
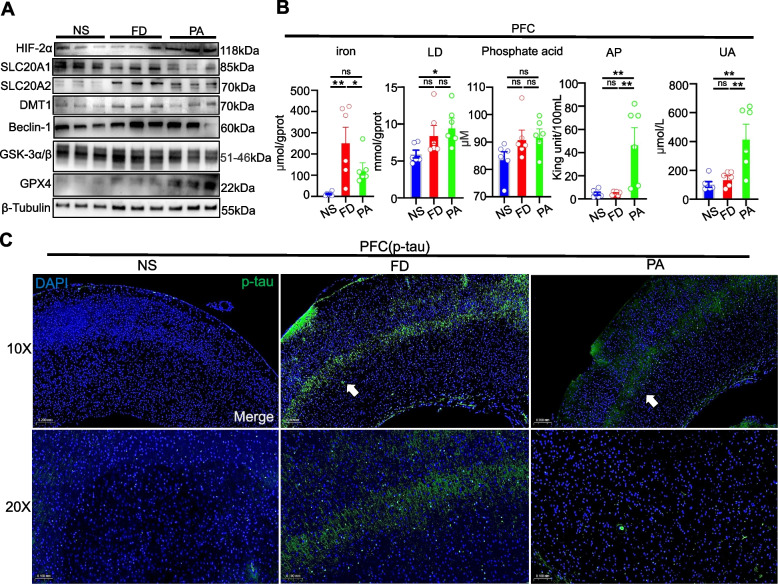
Effects of PA on cortical tissue under the HFe diet. **A** Western blotting showed the effects of PA on the expression levels of proteins involved in iron accumulation, cell death, AD risk makers and Pi transporters in the cortical tissue of the mice (3 mice each group). **B** Quantification of iron, LD, Pi, AP and UA levels indicated that PA counteracted the HFe-induced increase in levels of iron in cortical tissue but had no effect on cortical LD and Pi levels; in contrast, PA significantly increased AP and UA levels in cortical tissue compared to HFe-fed mice (above 5 mice each group).One-way ANOVA with post-hoc LSD test was used for significance. **C** Representative fluorescence sections of p-tau deposition and expression in cortical tissue in three groups by immunofluorescence (3 mice each group). Scale bar = 100 μm or 200 μm. All data are means ± SEM. Statistical significance vs. with the vehicle-only treatment group. *p < 0.05, **p < 0.01, ***p < 0.001, ns: no significant difference

The hippocampus and prefrontal cortex are the two main brain regions responsible for short- and long-term memory and decision-making functions [31]. We found that the prefrontal cortex of mice in the NS and PA groups exhibited decreasing expression of glycogen synthase kinase 3 beta (GSK-3β) compared to the FD group (Fig. 4A). Iron overload induced elevated levels of brain iron transporter DMT1 and brain HIF-2α, but PA intervention reduced DMT1 but not HIF-2α protein levels compared to HFe-fed mice. There was no difference in Beclin-1 expression involved in autophagy induction of tau secretion [32]. PA promoted expression of the ferroptosis factor GPX4 in prefrontal cortex. As expected, iron intake led to iron accumulation in the prefrontal cortex of FD mice, while PA intervention prevented the elevation of iron concentrations (Fig. 4B). In addition, transferrin was highly immunoreactive in the prefrontal cortex area of FD mice but levels were lowered in PA mice (Supplemental Fig. 2). Finally, PA enhanced AP activity and UA levels.

Considering the Pi transport, the HFe diet produced a decrease in slc20a1 protein content and an increase in slc20a2 protein content in the prefrontal cortex of the FD group, compared with NS mice, while PA resulted in decreased expression of slc20a1 and slc20a2 compared with FD mice (Fig. 4A). However, the Pi levels did not change among the different groups.

Substantial behavioral and imaging evidence implicates p-tau in cognitive functions [1]. We thus used immunostaining to detect p-tau protein content. In Fig. 4C, the HFe diet induced higher phosphorylation of tau, as reflected by a higher density of fluorescence. The higher phosphorylated area in FD prefrontal cortex was mainly distributed in prefrontal Layer V (Area indicated by white arrows in Fig. 4C). In contrast, PA significantly reduced the fluorescence of p-tau in the prefrontal cortex.

**Fig. 5:**
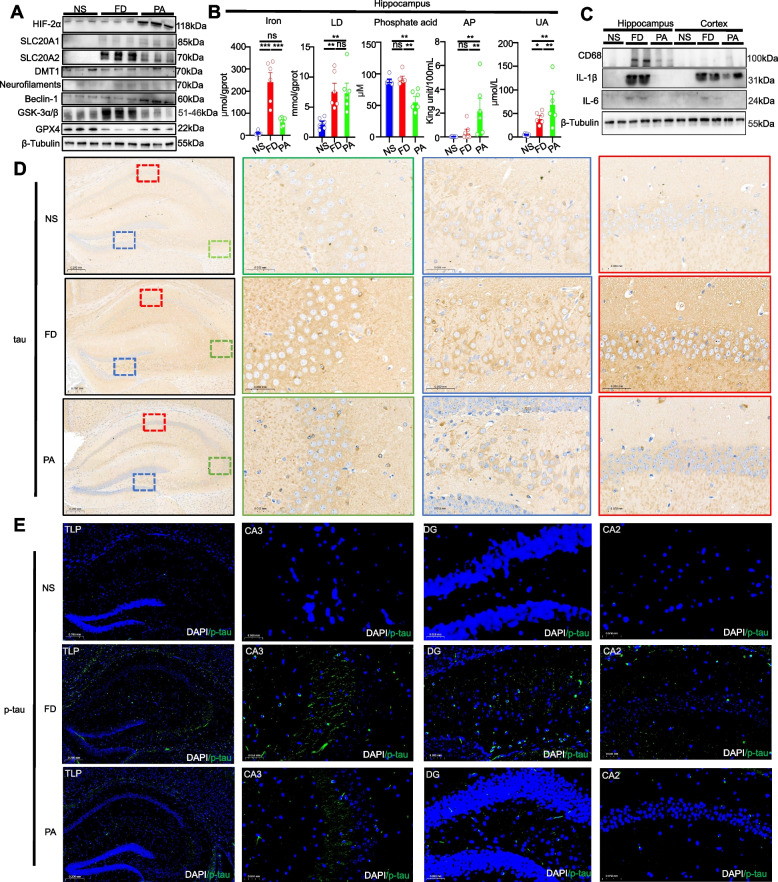
Effects of PA on hippocampal tissue under the HFe diet. **A** Western blots detecting protein levels involved in iron accumulation, cell death, AD risk makers and Pi transporters in hippocampal tissue of the mice (3 mice each group). **B** Quantification of iron, LD, Pi, AP and UA levels indicated that PA not only counteracted the HFe-induced increase in levels of iron (but not LD) in hippocampal tissue, but also decreased Pi and increased AP and UA levels in hippocampal tissue from the mice (above 5 mice each group).One-way ANOVA with post-hoc LSD test was used for significance. **C** Western blotting for detection of inflammatory protein levels indicated that PA counteracted the HFe-induced increase in protein levels of IL-6, IL-1β and CD68 in cortical regions and IL-6 and IL-1β in hippocampal regions (2 mice each group). **D** Representative IHC sections of tau deposition and expression in different areas of hippocampal tissues from the NS, FD and PA groups (3 mice each group). Green, red and blue dotted box arrowheads indicate CA3, CA2 and DG areas, respectively. Scale bar = 500 μm or 200 μm. **E** Representative sections of p-tau deposition (green) in the entire hippocampus, and in the CA3, DG and CA2 areas of the NS, FD, PA groups by immunofluorescence. The PA group showed a lowering of fluorescence intensity compared to the FD group (3 mice each group). Scale bar = 500 μm or 200 μm. All data are means ± SEM. One-way ANOVA with post-hoc LSD test was performed in (E). *p < 0.05, **p < 0.01, ***p < 0.001, ns: no significant difference

Likewise, the hippocampus exhibited decreased expression of GSK-3β in the NS and PA groups compared to FD animals (Fig. 5A). After PA oral gavage, ferroptosis factor GPX4 protein levels increased in the hippocampus of HFe-fed mice to levels close to those observed in NS mice. Moreover, PA intervention significantly reduced the beclin-1 content but not the HIF-2α protein levels compared to the hippocampus of the FD groups (Fig. 5A). There was no difference in expression of DMT1 involved in iron transport. The protein levels of Pi transporter slc20a1 and slc20a2 increased under the HFe diet, with a decrease in slc20a2 protein in the PA hippocampus compared to the FD hippocampus (Fig. 5A). Hippocampal iron accumulation was promoted under the HFe diet and was significantly reduced by PA administration. The metabolic data showed that PA could significantly decrease Pi and increase alkaline phosphatase and uric acid levels in the hippocampus of FD mice but did not significantly alter LD levels, even though HFe induced a high LD level in the hippocampus (Fig. 5B).

Understanding the inflammatory mechanisms involved in LCN2-mediated BBB damage may be key in the development of cognitive dysfunction [33]. Importantly, inflammatory IL-1β and IL-6 were increased under the HFe diet, and PA decreased CD68 protein in the hippocampus compared to the FD hippocampus, but we observed non-significant changes of CD68 in the prefrontal cortex (Fig. 5C). Furthermore, immunostaining for tau and p-tau proteins revealed that tau and p-tau immunoreactive areas were enhanced in CA1, CA3 and DG layers in the hippocampus of FD mice compared with the NS mice, while PA gavage downregulated the tau protein-immunoreactive accumulations and in particular abolished p-tau immunoreactivity (Fig. 5D and E).

**Fig. 6:**
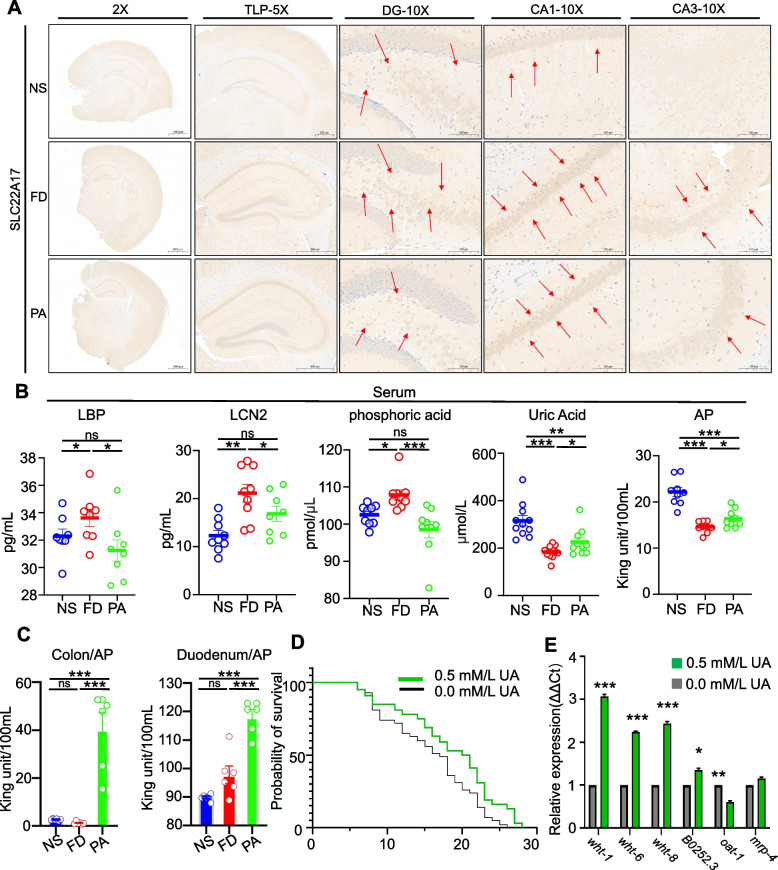
SLC22A17 deposition in the hippocampal area and AP-UA axis regulated by PA. **A** Representative IHC sections of SLC22A17 deposition and expression compared in the hippocampal areas in three groups (3 mice each group). Red arrowheads indicate SLC22A17 immunoreactive areas. Scale bar = 500 μm. **B** Quantification of iron, LD, Pi, AP and UA levels indicated that PA not only counteracted the HFe-induced increase in levels of LBP, LCN2 and phosphate acid, but also increased levels of UA and AP in serum from the mice (above 8 mice each group). One-way ANOVA with post-hoc LSD test was used for significance. **C** Quantification of AP activity indicated that PA significantly increased AP activity in the colon and duodenum of mice (above 5 mice each group).One-way ANOVA with post-hoc LSD test was used for significance. **D** Lifespan extension for *C.elegans* VH254 fed with *E. coli* OP50 after adding 0.5 mM UA compared to controls (0 mM UA) from the L4 developmental stage maintained at 15 °C (120 worms per group).One-way ANOVA with post-hoc LSD test was used for significance. **E** mRNA expression of UA transport genes of worms from the p-tau control and UA-treated group (600 worms per group).One-way ANOVA with post-hoc LSD test was used for significance. All data are means ± SEM. Statistical significance: *p < 0.05, **p < 0.01, ***p < 0.001, ns: no significant difference

Several studies have indicated that the iron-binding protein LCN2/receptor SLC22A17 system can effectively transport iron to outcompete the iron utilization of macrophages and prevent brain iron accumulation [12]. Brain sections revealed that FD mice showed pronounced activation of LCN2 receptor SLC22A17 in CA1, CA3 and DG areas of the hippocampus (Fig. 6A), indicating a higher iron-transporting effect in FD mice. Moreover, TF was highly immunoreactive in the CA1 and PC areas of the FD group (Supplemental Fig. 3). Lastly, the circulating LBP, LCN2 and Pi levels were significantly downregulated by PA intake compared to FD mice, whereas they did not significantly change between the NS and PA mice, confirming the reduced iron overload and metabolic toxicity with PA (Fig. 6B). However, serum AP and UA levels were significantly reduced by the HFe diet whereas PA significantly promoted the AP and UA levels (Fig. 6B). Consistent with brain data, higher AP activity induced by PA was observed in the duodenum and colon (Fig. 6C). The lifespan-extending effect of PA was verified by UA in tau-expressing worms (*p* < 0.01, *n* = 120), indicating that UA plays a beneficial role in toxic tau alleviation (Fig. 6D). Additionally, UA upregulated *wht1*, *wht6* and *wht8* and decreased *oat-1* expression in p-tau-transgenic nematodes (Fig. 6E).

**Fig. 7:**
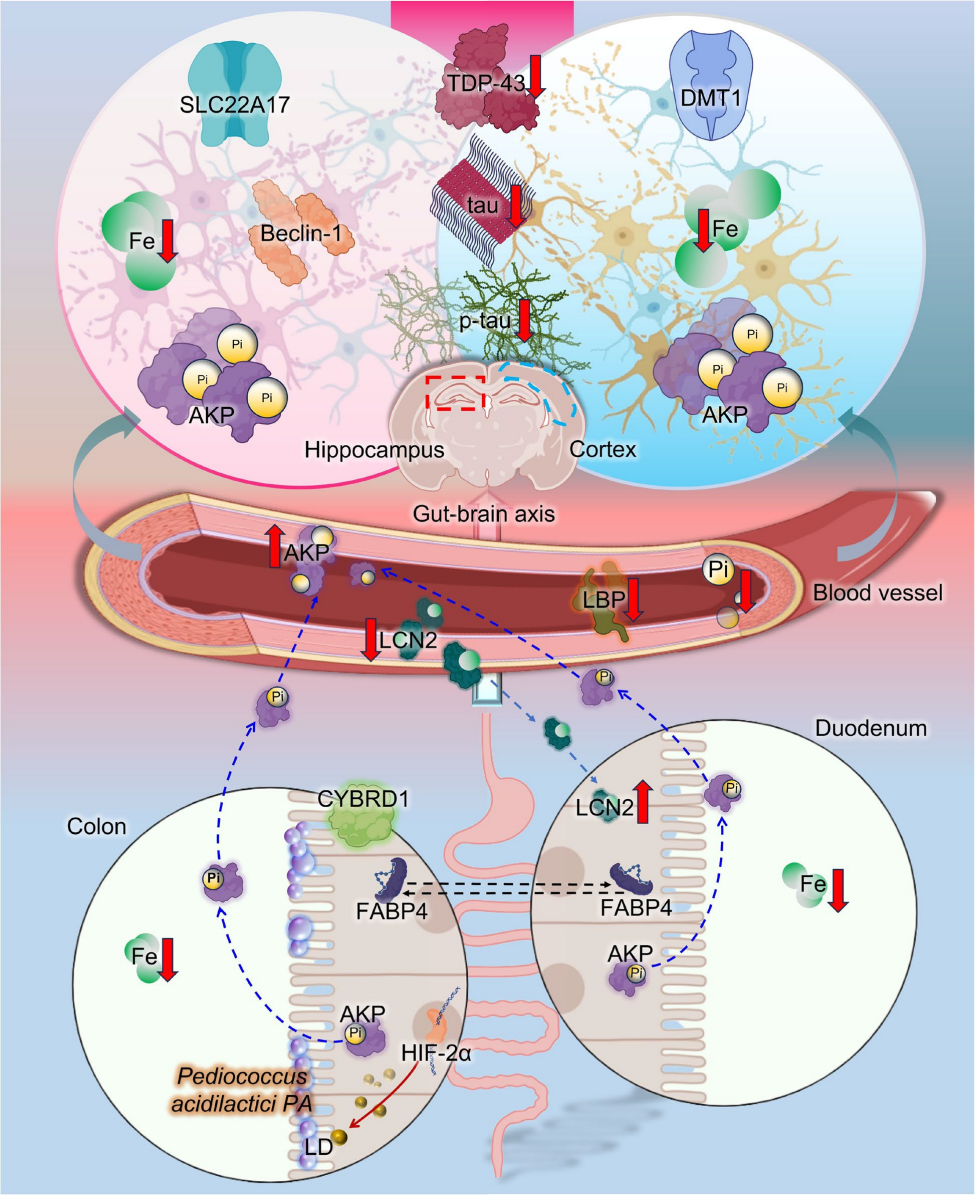
A proposed working model showing how the probiotic PA prevents cognitive impairment

These results provide strong support and new insights for the effect of iron overload and the probiotic PA as a novel iron modulator throughout the body (Fig. 7). The schematic illustrates the important roles of microbial AP and circulating metabolites (Pi and UA) to manipulate host systemic iron homeostasis. Given the finding that gut iron levels are sensed by intestinal probiotic *L. reuteri* [10], we broadened the scope of our study to include brain iron accumulation and cognitive behavior influenced by PA intervention. However, brain iron levels were not notably affected by HIF-2α signaling. PA regulated AP with strong expression from intestine to brain, and we discovered the probiotic-mediated iron-transporting axis with duodenum/slc22a17-circulation/Lcn2-prefrontal cortex/Dmt-1. Accompanied by the above results, the reduction of Pi in the circulation and brain can potentially lead to decreased inflammation and neurotoxicity, whereas UA promotion in the brain can contribute to cognitive improvement. To summarize, these responses can lead to decreased iron accumulation and p-tau levels after PA intervention, which can potentially be further investigated for their role in AD pathogenesis.

## Discussion

Our cross-species analysis from *C. elegans* to mice provides evidence that PA intake has a critical role in cognitive dysfunction. *Pediococcus acidilactici*, previously found a commensal in human beings [34], has been proved to have beneficial effects in improving spatial learning and memory mimicking AD in adult zebrafish and also already to reduce the content of heavy metals (copper/ nickel) in blood of humans [35, 36]. The BN did not exhibit a benefit on spatial learning and memory. For decades, A-β and tau have been at the center of therapeutic targets for AD, while TDP43 and OPN have also demonstrated potential [37]. Diverse studies have supported the hypothesis that microbiota interventions could reduce aggregation of tau in the hippocampus of AD model mice [38]. Our results suggest that an HFe diet causes cognitive dysfunction in mice, which is associated with increased expression of APP, TAU, TDP43 and OPN in the prefrontal cortex and hippocampus. In order to obtain the most similarity baseline of mice and avoid diverse differences of mice with old age [39], we enrolled 8-week age adult mice not aged mice for HFe diet fed. Supplementation with PA resulted in the reduction of tau levels and hyperphosphorylated tau whereas APP and OPN remained unchanged in the HFe-fed AD mice. We also saw evidence for this in transgenic p-tau *C. elegans*.

The first targeted site of probiotics is the intestine. PA can enter the intestine with viable bacteria due to its favorable acid and bile salt tolerence. The different segments of intestine have distinct physiological functions, particularly the duodenum and colon [40]. Previous research found that duodenum but not colon senses iron ions in the intestine and mainly takes up iron via HIF-2α/Cybard1 signaling [41], which could be regulated by *Lactobacillus reuteri*. Consistently, PA was found to influence iron distribution by inhibiting duodenum Cybard1. Similar to lactic acid bacteria, PA can also produce a great deal of lactic acid in the gut and thereby stimulate HIF-2α signaling. LD is not only required for intestinal pH homeostasis, but is also essential for the regulation of brain energetic metabolism, neural cell repair and function of the BBB [42, 43]. However, we observed that PA failed to significantly regulate brain HIF-2α, suggesting it is not the master regulator of brain iron homeostasis.

Our findings extend the gut-brain axis hypothesis of iron accumulation through the *slc22a17*/*lcn2* and AP-uric acid linkage from gut to brain. We observed that iron transport driven by PA throughout the body connects the duodenum-brain axis. Despite different transporters, iron was transported from the duodenum (*slc22a17*) to the circulation (*lcn2*), from which it was taken up in the prefrontal cortex by *dmt-1*. Consistent with this hypothesis, a previous study also revealed that microbiota transplantation improves behavioral impairment by promoting fecal *lcn2* in AD mice [40]. However, colonic iron transport was unaffected since two main transporters (*lcn2* and *dmt-1*) were not changed by HFe or PA intake. Interestingly, the difference between the duodenum and colon is reflected in FABP4 and other resistant systems. We observed that FABP4 was elevated in the duodenum and reduced in the colon by PA, suggesting the existence of a transport system for lipids from duodenum to colon in which the transporting substance is the required next step. Our data similarly revealed that probiotic PA could enhance colon FABP4 expression, lowering circulating LBP levels, and thereby reduce inflammatory IL-1β, IL-6 and microglial CD68 expression in the brain. This was inversely correlated with the results of a previous microbiota transfer study [44]. Moreover, phosphoric acid intake can be downregulated by FABP4 expression via fatty acid synthesis [45], which indicated that PA intervention lowered circulating phosphoric acid levels in HFe mice.

For the AP and related metabolic changes in the gut-brain axis, PA administration induced higher AP activity from gut to blood and brain. Three aspects may be involved in altering PA-mediated effects which alleviate cognitive impairment: (i) In the gut, the PA-mediated increase in AP activity also improved barrier integrity as reflected by reduced serum LBP concentration in response to intestinal bacterial lipopolysaccharide leakage into the circulation [46]. Interestingly, high-iron intake induced the intestinal barrier damage and leading to a protective effects of higher ZO-1 expression to the host barrier. PA counteracted the high-iron induced Zo-1 elevation to the normal level and prevented the intestinal iron damage. In contrast, BN failed to maintain intestinal barrier integrity which developed into colitis, suggesting an impaired gut-brain axis. Thus, PA might reduce systemic inflammation to relive further brain inflammation via AP elevation; (ii) In the brain, AP dephosphorylated the toxic hyperphosphorylated tau protein in the central nervous system [47], and was upregulated by PA, which also contributed to reducing tau phosphorylation. Consistently, AP (Akp3)-deficient and hyperphosphatemia mouse models have confirmed that AP activation leads to a decrease in serum and brain Pi levels and an improvement in tau-induced neurocognition [48, 49]. Thus, decreased phosphate levels by PA intake may delay the hyperphosphorylation process and influence the formation of phosphorylated tau protein; (iii) In the brain, higher levels of UA, which is one of the purine metabolites that possesses antioxidant properties, may exert neuroprotective actions in AD [50]. A previous study has shown that brain UA injection suppressed microglial inflammation and exerted neuroprotective effects in a neurodegenerative disease model [51]. Thus, high brain UA levels in PA-treated mice may protect against cognitive dysfunction. Additionally, PA enhanced UA transport by maintaining high-capacity UA with *wht8* and decreasing the UA efflux activity of *oat-1*.

It has been reported that p-tau is a complex mixture and there exist an ordered phosphorylation process and a balance in the phosphorylation of tau [21]. According to our investigation, both tau-expressing *C.elegans* and the HFe animal model exhibited a tau-reducing effect after PA intake. Mechanical exploration in worms showed an increase in transcription of genes responsible for autophagy, mis-folded tau degradation and tau dephosphorylation. In addition, a 2-fold decrease in molecular motor genes for tau axonal transport by PA indicated that PA altered the clearance of tau and prevented tau toxicity in tau-expressing *C.elegans*. In addition, GSK-3β, one of the kinases involved in tau hyperphosphorylation [52], was downregulated in mice by PA intake. PiT1 (SLC20a1) and PiT2 (SLC20a2), which are required for brain phosphate transport [48], were strongly expressed in mouse prefrontal cortex and hippocampus, suggesting that there are brain-area-specific differences in phosphate transport. PiT1 is mainly responsible for the prefrontal cortex and PiT2 was influenced by iron overload in the hippocampus, but how they modulate PiT1 and PiT2 expression is not fully understood.

## Conclusions

Together, our data provide a more comprehensive understanding of the iron-overload contributing to AD risk. Our dietary PA manipulation studies demonstrate that PA is an important mediator of tau protein reduction and AP activation. PA improves iron retention by affecting LD-mediated HIF-2α signaling in the colon but not in the brain, thus affecting LCN2 in the duodenum and brain. Increased systemic AP activity in the digestive tract reduced systemic Pi levels and tau phosphorylation in the brain (Fig. 7). We suggest that an expansion of both the duodenum-brain transport axis and colon-brain AP axis underlies the mechanism by which AD affects tau protein clearance and inflammatory pathways. These results may provide important insights into the pathobiology of AD and associated complications.

## Methods

### Strains of Bacteria and *C. elegans*

The two bacterial strains PA and BN were used as materials to evaluate their function in ameliorating HFe-induced cognitive impairment. The two strains were isolated from naturally fermented pickles and fermented soybean paste, respectively. These strains were preserved in the China General Microbiological Culture Collection Center (CGMCC; *Pediococcus acidilactici* CGMCC No. 24714 and *Bacillus subtilis* CGMCC No.15635) and were cultured in de Man, Rogosa and Sharpe broth (Oxoid, Hampshire,UK) at 37 °C for 18 h. The fermented broth was collected and dried as bacterial powder. Freeze-dried bacteria were prepared for gavage using a freeze-dryer (Christ α-2-4LD plus, Germany).

*Escherichia coli* (*E. coli*) strain OP50 was obtained from the Institute of Rate Biophysics of Chinese Academy of Sciences and cultured in Luria–Bertani medium (Oxoid) at 37 °C for 18 h.

The tau transgenic *C.elegans* strain VH254 [pha-1 (e2123) III; hdEx81)], expressing FLAG-human pseudohyperphosphorylated tau, was purchased from the Caenorhabditis Genetics Center (University of Minnesota, USA). VH254 strain was cultured at 25 °C on Nematode Growth Medium (NGM) plates using living *E. coli* strain OP50 as food.

### In vitro acid resistance and bile salt tolerance of probiotics

Bacterial strains were subcultured for 3 generation before test. Then the potential probiotics were screened using simulated gastric juice (pH 2.5, 3 h of incubation) and 0.3%(w/v) solution of bile salts as described previously [53].

### Longevity assays in *C.Elegans*

The VH254 strain was cultured on NGM plates containing cholesterol and a lawn of greater than 3.0 × 10^9^ CFU/ml of *E. coli* OP50 cells at 25 °C. Once the worms had reached the L4 stage, they were picked up and placed onto new plates with the same magnitude order of *E. coli* OP50 or PA (3.2 × 10^8^ CFU/ml). Worms were transferred to new plates every 2 days, and the days alive were calculated as previously described [18]. For assessment of the urate effect on tau-expressing worms, L4 stage animals were placed onto new *E. coli* OP50 plates with and without 0.5 mM urate, and the days alive were recorded. This experiment was repeated at least two independent times.

### Measurement of behavioral assays in *C.elegans*

Pharyngeal pumping analysis was performed on day 3, 6 and 9 of adulthood by counting the number of pharynx rear bulb movements at 25 °C [18]. The results are presented as pumping rates in a period of 30 s. For assessment of oscillating rate, the worms were transferred to NGM plates without lawns on day 3 and 6 of adulthood and the number of head swings were counted in 30 s. Head swing behavior was defined as a change in direction with a change angle greater than 90 degrees. On the fourth day of incubation at 20 °C [18], the body bending rate was analyzed. For body bend assessment, the worms were transferred to NGM plates without bacterial solution, and each covered by a drop of M9 buffer (Solarbio, Beijing). After 10 seconds of adaptation, the number of body bends in 30 s at room temperature was recorded. Three independent trials of the above tests were performed with 10 worms for each bacterial species.

### Animals, housing and experimental design

The protocol using experimental animals was performed in strict accordance with the recommendations in the Guide for the Care and Use of Laboratory Animals by the National Institutes of Health at the University of Science and Technology Beijing (BLARC 2019A084).

C57BL/6 J mice were purchased from Huafukang Biotechnology Company (China) and maintained in the Center for Animals in the General Hospital of the Chinese Armed Police Forces. The 8-week-old male mice were randomly distributed into four groups (NS, normal saline group; FD, high-Fe diet group; PA, PA strain intervention group; BN, BN strain intervention group), and housed with sterile food and water under standard controlled conditions (12-h light/dark cycle; temperature of 22 °C ± 2 °C; humidity 55% ± 5%). Each group contained 10 mice and five mice were housed per cage.

In this experiment, the FD group and probiotic-treated groups were fed a sterile high-Fe diet (TP0452M, 600 ppm of ferric citrate, Trophic Animal Feed High-tech Co., Ltd., China), compared to the NS group fed control chow feed (TP0452, 45 ppm of ferric citrate, Trophic) for 60 days. Mice were checked for body weight and diet intake every 3 days.

The probiotic treatments began in the first week. Two intervention groups were given the probiotics BN or PA, at a daily dose of 1.0 × 10^9^ CFU per mouse. The mice in the NS and FD groups were fed an equivalent volume of saline daily.

### Barnes and Y-mazes tests

Barnes mazes were used to assess spatial memory 60 days after probiotics administration by three blinded observers. The maze consisted of a white circular table (Ø 150 cm) with 20 perimetric holes, one of which was connected to a hidden escape box. More details are presented in a previous study [54]. Black curtains equipped with spatial cues (simple geometric symbols) surrounded the maze. The mice were trained at 10 a.m. every day for 7 days. Each mouse was allowed 3 min to find the escape box.

The Y-maze test was performed 60 days after probiotics administration by three blinded observers to assess short-term memory. There were three arms in the maze at angles of 120°, each arm measured 30 cm × 8 cm × 15 cm (length × width × height) with a removable divider at the center. More details are described in a previous study [27]. Each mouse was allowed 3 min to develop short-term memory with the divider in place, which was then removed to restore the maze to the three open-arms state. After 2 h, the new arm was opened, and the mice were allowed to move freely in the three arms for 3 min.

The sessions were video recorded and analyzed by SuperMaze software (XinRuan Information Technology Co., China). Time (s) and moving distance (cm) before locating the escape hole were calculated. In the first trial, velocity (cm/s) was calculated as a measure of locomotor activity.

### Hematoxylin and eosin (H&E) staining

Intestinal tissues were collected and fixed in 10% (v/v) buffered formaldehyde for 48 h. Then the tissue sections were stained using H&E staining as described previously [55].

### RNA extraction and qPCR

After a 9-day treatment with PA or *E. coli* OP50, the VH254 worms (*n* = 600) in each group were washed with M9 buffer and lysed in TRIzol reagent (Life Technologies, USA). For mice, colon and duodenum tissues were collected and homogenized in TRIzol (*n* = 10). RNA was extracted, purified and evaluated using a Nanodrop ND-1000 Spectrophotometer (Thermo Fisher Scientific, USA). cDNA was reverse-transcribed using a QuantScript RT Kit (KR103, TIANGEN Biotech, China), and the ABI system (Quant Studio 5, USA) was used to perform qPCR following the instructions of a commercial TB green Master reagents kit (RR820A,TaKaRa, Japan). Specific gene primers are shown in Supplemental Table 1. The relative quantification of mRNA expression was calculated using the 2^-ΔΔCt^ method [10], normalized to the expression of TBA-1 (tubulin, worms) or GAPDH (mice).

### Western blotting

Proteins were extracted from worms (*n* = 700 per group) and mouse tissues using TPER tissue lysis buffer (Thermo Scientific) supplemented with a protease inhibitor cocktail (Roche Diagnostics,Switzerland). The samples, containing 30 μg protein each, were resolved by 10–15% polyacrylamide gel electrophoresis and transferred onto PVDF membranes (0.22 μm, Millipore, USA). Membranes were blocked with 5% powdered skim milk for 1 h on a gentle shaker. The membranes were then incubated with primary antibodies (1:1000, Supplemental Table 1) overnight at 4 °C followed by diluted horseradish peroxidase-conjugated secondary antibody (1:10000, Cell Signaling Technology, USA). Protein bands were visualized and quantified using chemiluminescence with the Tanon Blotting Detection System (Tanon, China).

### Immunofluorescence and immunohistochemical staining

The right hemicerebrum were fixed in 4% paraformaldehyde at 4 °C for 48 h and embedded in OCT compound (Yinghua, China) at − 80 °C. For p-tau staining, a CM1950 cryostat (Leica, Germany) was used to prepare the sagittal or coronal brain frozen sections at a thickness of 30 μm. After sectioning, the brain slices were mounted on Superfrost plus gelatin-coated glass slides (Thermo Fisher Scientific) and stored at − 80 °C. p-tau antibody (1:1000; Cell Signaling Technology) and Alexa Fluor 488–conjugated secondary antibody (1:500; Invitrogen, USA) were used for immunofluorescence staining in the dark on ice (Supplemental Table 2). Nuclei were stained with DAPI (4′6-diamidino-2-phenylindole, Beyotime, China). Images were acquired using a confocal Leica TCS SP8 microscope (Germany).

Frozen sections of hemicerebrum with a thickness of 15 μm were also used for immunohistochemical staining. Antigen retrieval was performed with Quick Antigen Retrieval Solution (Beyotime). Then the sections were incubated overnight at 4 °C with primary antibody (1:200–1:500, see Supplemental Table 2). After washing in TBST for three times, the sections are incubated with biotin-labelled secondary antibody (Supplemental Table 2) for 1 h at 37 °C. Immunoreactivity was determined using an HRP assay kit (Beyotime) according to the manufacturer’s protocol and was visualized under the Leica IIC50W microscope.

### Quantification of LBP, UA, LCN2 and AP in serum and tissues

Serum was centrifuged and collected from the blood at the end of the experiment for further studies. Serum was used for ELISA kits analysis (all included in Supplemental Table 3). Tissue samples from the mice, including colon, duodenum, hippocampus and prefrontal cortex, were analyzed and processed in accordance with the kit instructions (Supplemental Table 3).

As previously described [56], AP activity of mouse tissue homogenates was quantified using a commercial AP assay kit measuring 520 nm absorbance of red quinone derivatives formed from AP-mediated phenol production reacting with 4-aminoantipyrine and potassium ferricyanide in alkaline solution (Nanjing Jiancheng Bioengineering Institute, China).

### Statistical analysis

Results are expressed as means ± SEM. Significance between two groups was tested using a pair-wise t-test. Significance among multiple groups was tested using One-way ANOVA with post-hoc LSD test. GraphPad Prism 9.0 (Graphpad Software, USA) was used to conduct statistical analyses. Statistical significance is described in the figure legends as: * *p* < 0.05, ** *p* < 0.01, *** *p* < 0.001, ns, no significant difference.

### Supplementary Information

**Additional file 1: Supplemental Fig. 1.** (A) Quantification of histological colitis scores in the four groups (8 mice each group).One-way ANOVA with post-hoc LSD test was used for significance. (B) The speed of mice in the Barnes Maze and Y Maze. One-way ANOVA with post-hoc LSD test was used for significance. (C) Western blotting for detecting the protein levels of tau and p-tau in the hippocampus and cortical tissue of the mice (5 mice each group). (D) Exhibition of behavior including pumping rates, head swing rates and body bending rates in PA- or *E. coli* OP50-treated *C.elegans* VH254.Paired t-tests were used for significance. (E) Quantification of immunofluorescence intensity for p-tau proteins. Paired t-tests were used for significance. (F) In vitro acid resistance of PA. (G) In vitro bile salt tolerance of PA. **Supplemental Fig. 2.** Representative IHC sections showing that PA decreased TF, TAU and slc22a17 expression in the brain prefrontal cortex regions of the three groups. **Supplemental Fig. 3.** Representative IHC sections of TF deposition in brain hippocampal regions of the three groups. **Supplemental Table 1.** Primers used for qRT-PCR analysis. **Supplemental Table 2.** Antibodies used in this study. **Supplemental Table 3.** ELISA kits and biochemical kits used in this study.

## Data Availability

All data generated during this study are included Supplemental Figs. 1, 2, 3 and Supplemental Tables 1, 2, 3.

## Abbreviations

AD: Alzheimer‘s disease
AP: Alkaline phosphatase
APP: β-amyloid precursor protein
ATP: Adenosine triphosphate
BBB: Blood-brain barrier
BN: *Bacillus subtilis* CGMCC15635
CA1: Cornu ammonis 1
CA3: Cornu ammonis 3
CD68: Macrophage Antigen CD68
CFU: Colony forming units
cyb561: Cytochrome B561
CYBRD1: Cytochrome B Reductase
DAPI: 4′,6-diamidino-2-phenylindole
DG: Dentate gyrus
DMT1: Divalent metal transporter 1
eIF2α: Eukaryotic Translation Initiation Factor 2A
ELISA: Enzyme linked immunosorbent assay
FABP4: Fatty Acid Binding Protein 4
FD: High iron-diet feed
FGF23: Fibroblast Growth Factor 23
Fpn: Ferroportin
GPX4: Glutathione Peroxidase 4
GSK-3α/β: Glycogen Synthase Kinase-3 Alpha/Beta
H&E: Hematoxylin and eosin
Hamp: Hepcidin Antimicrobial Peptide
HFe: High-Fe diet
Hgb: Histoglobin
HIF-2α: Hypoxia Inducible Factor 2 Subunit Alpha (HIF-2α)
HO-1: Heme Oxygenase 1
Hpx: Hemopexin
HRP: Horseradish peroxidase
IHC: Immunohistochemistry
IL-1β: Interleukin 1 Beta
IL-6: Interleukin 6
LBP: Lipopolysaccharide Binding Protein
LCN2: Lipocalin 2
LD: Lactic acid
LPS: Lipopolysaccharide
mRNA: messenger Ribonucleic Acid
MRS: de Man, Rogosa and Sharp
muc-1: Mucin 1
NS: Normal saline
OPN: Osteopontin
PA: *Pediococcus acidilactici* PA
Pi: Phosphoric acid
PiT1: Phosphate Transporter 1
PiT2: Phosphate Transporter 2
qRT-PCR: quantitative Reverse transcription polymerase chain reaction
SLC: Solute carrier
SOD2: Superoxide Dismutase 2
TAU: Microtubule-associated protein tau
TDP-43: TAR DNA binding protein 43
TF: Transferrin
TLP: Trisynaptic circuit
TNAP: Tissue nonspecific alkaline phosphatase
trRC: Transferrin Receptor
UA: Uric Acid
WT: Wild-type
ZO-1: Tight Junction Protein 1
