# Supplementary material for: Pediococcus acidilactici reduces tau pathology and ameliorates behavioral deficits in models of neurodegenerative disorders
Source: Cell Commun Signal. 2024 Jan 30;22:84. doi: 10.1186/s12964-023-01419-3 (PMC10826277; doi:10.1186/s12964-023-01419-3)
Supplement: Supplementary file 2 — Additional file 1: Supplemental Fig. 1. (A) Quantification of histological colitis scores in the four groups (8 mice each group).One-way ANOVA with post-hoc LSD test was used for significance. (B) The speed of mice in the Barnes Maze and Y Maze. One-way ANOVA with post-hoc LSD test was used for significance. (C) Western blotting for detecting the protein levels of tau and p-tau in the hippocampus and cortical tissue of the mice (5 mice each group). (D) Exhibition of behavior including pumping rates, head swing rates and body bending rates in PA- or E. coli OP50-treated C.elegans VH254.Paired t-tests were used for significance. (E) Quantification of immunofluorescence intensity for p-tau proteins. Paired t-tests were used for significance. (F) In vitro acid resistance of PA. (G) In vitro bile salt tolerance of PA. Supplemental Fig. 2. Representative IHC sections showing that PA decreased TF, TAU and slc22a17 expression in the brain prefrontal cortex regions of the three groups. Supplemental Fig. 3. Representative IHC sections of TF deposition in brain hippocampal regions of the three groups. Supplemental Table 1. Primers used for qRT-PCR analysis. Supplemental Table 2. Antibodies used in this study. Supplemental Table 3. ELISA kits and biochemical kits used in this study. [file 12964_2023_1419_MOESM1_ESM.zip › Supplementary/WB bands.pptx]

## Slide 1
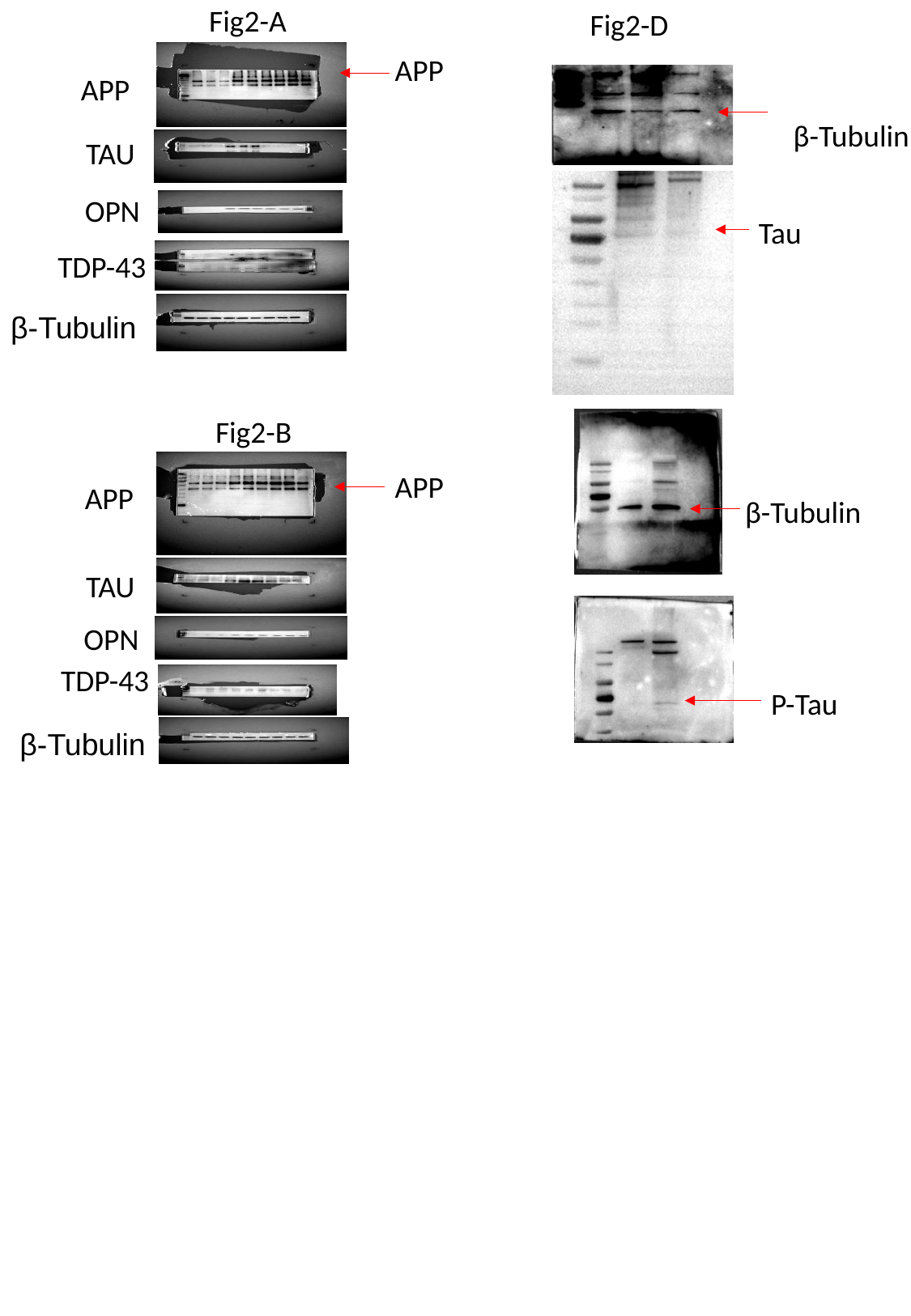

Fig2-D
Fig2-A
APP
APP
β-Tubulin
TAU
OPN
Tau
TDP-43
 β-Tubulin
Fig2-B
APP
APP
β-Tubulin
TAU
OPN
TDP-43
P-Tau
 β-Tubulin

## Slide 2
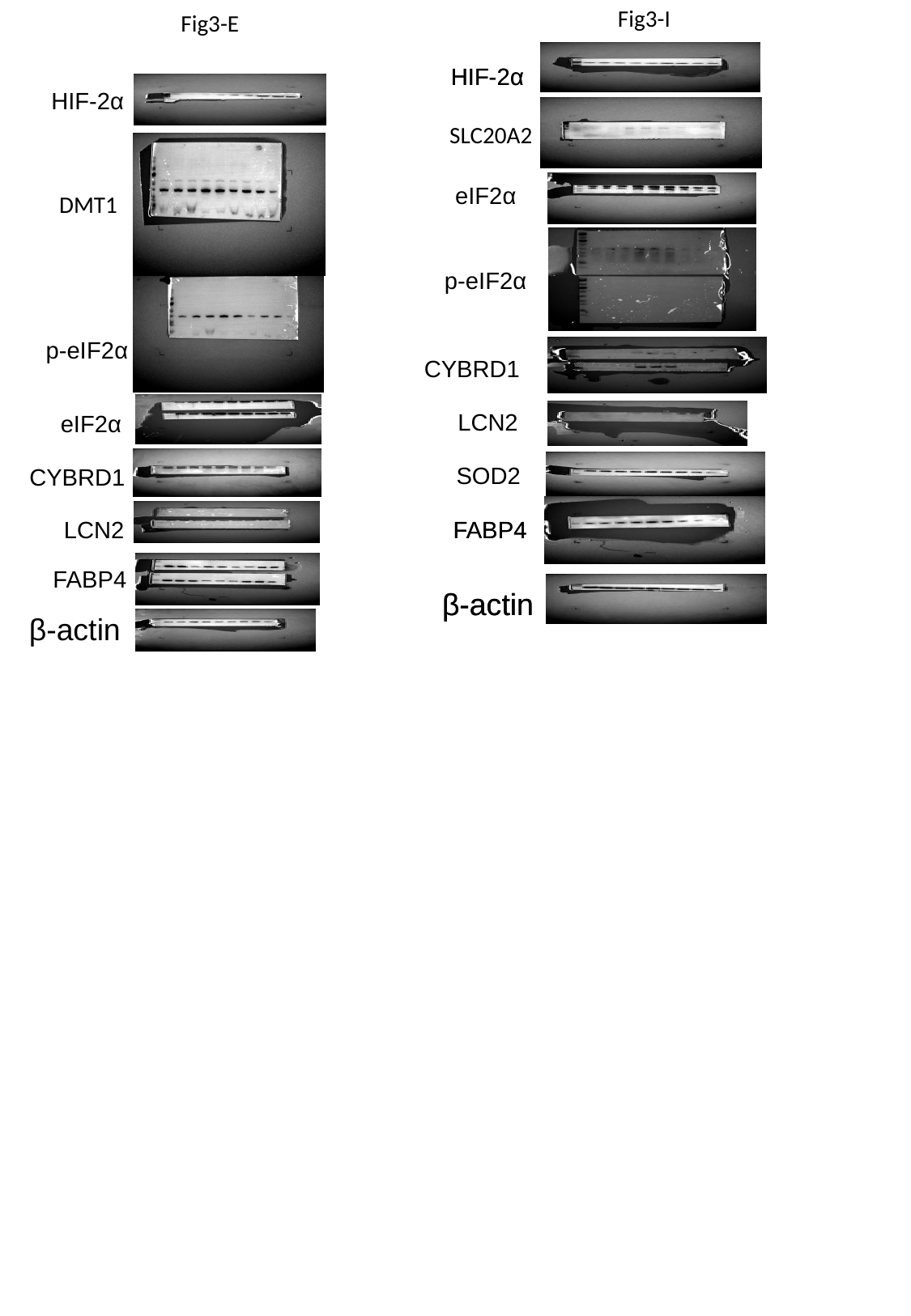

Fig3-I
Fig3-E
 HIF-2α
 HIF-2α
 HIF-2α
SLC20A2
 eIF2α
DMT1
 p-eIF2α
 p-eIF2α
 CYBRD1
 LCN2
 eIF2α
 SOD2
 CYBRD1
 LCN2
 FABP4
 FABP4
 FABP4
 β-actin
 β-actin
 β-actin

## Slide 3
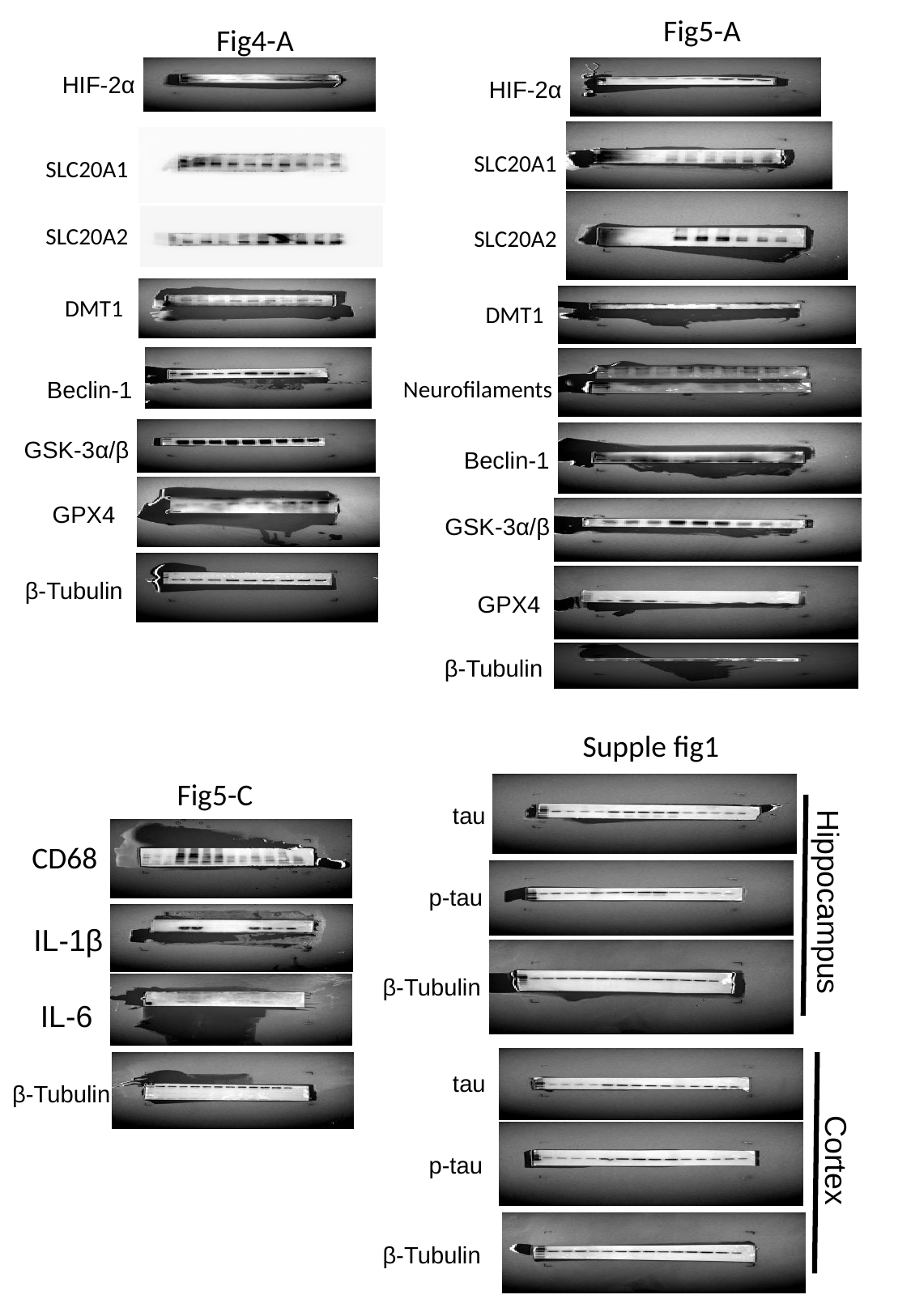

Fig5-A
Fig4-A
 HIF-2α
 HIF-2α
SLC20A1
SLC20A1
SLC20A2
SLC20A2
DMT1
DMT1
 Beclin-1
Neurofilaments
 Beclin-1
GSK-3α/β
 GPX4
GSK-3α/β
 β-Tubulin
 GPX4
 β-Tubulin
Supple fig1
Fig5-C
tau
CD68
p-tau
Hippocampus
 IL-1β
β-Tubulin
IL-6
 β-Tubulin
tau
Cortex
p-tau
β-Tubulin
